# A rare aggravation of severe mucositis post chemotherapy in a child with acute lymphoblastic leukemia

**DOI:** 10.12688/f1000research.2-196.v1

**Published:** 2013-09-24

**Authors:** Adlette Inati, Grace Akouri, Hussein A Abbas

**Affiliations:** 1 School of Medicine, Lebanese American University, Byblos, Lebanon; 2 Division of Pediatric Hematology Oncology, Rafic Hariri University Hospital, Beirut, Lebanon; 3 Faculty of Medicine, American University of Beirut, Beirut, Lebanon

## Abstract

Oral mucositis is a debilitating manifestation in children undergoing chemotherapy and radiotherapy. Children with mucositis should be properly managed in order to prevent further exacerbation and adverse complications. We hereby present the first report of a severe chemotherapy-induced mucositis, plausibly aggravated by improper dental hygiene leading to shedding of the ventral part of the tongue in a child with pre-B acute lymphoblastic leukemia (ALL). The patient steadily and gradually recovered her oral maneuvers and ability to speak several months later. Her tongue underwent hypertrophy as a compensatory mechanism. We recommend that critical and regular assessment of the oral mucosa and proper dental care and oral hygiene be emphasized in all pediatric patients receiving chemotherapy. Families of affected children need to be educated about the benefits and modes of optimal oral hygiene for their children and the need to seek immediate care for mouth pain and or lesions. Optimal treatment for mucositis needs to be instituted without delay in this high risk pediatric population. Such a preventive and therapeutic approach may prevent associated life threatening oral and systemic complications, promote rapid and complete mucosal healing, alleviate pain and improve quality of life in children with cancer.

## Case report

We report a 5-year old female with average risk (AR1) pre-B acute lymphoblastic leukemia (ALL) who presented with severe oral mucosal pain and fever of 38.7 ^o^C on day 22 induction (EORTC Children’s Leukemia Group protocol AR1 shown in Table 1).

**Table 1.** EORTC Children’s Leukemia Group Protocol AR1.

**Drugs**	**Dose**	**Route**	**Number of days/doses**	**Days**
**Methotrexate**	12 mgs	IT	1	1
**Prednisone**	60 mg/m2/day	PO	28	1 to 28
**Vincristine**	1.5 mg/m2	IV	4	8,15,22 and 29
**Daunorubicin**	30 mg/m2	IV	4	8,15,22 and 29
**L-Asparaginase**	10,000 U/m2	IM	8	12,15,18,22,25,29,32 and 35
**Methotrexate** **Hydrocortisone** **Cytosine Arabinoside**	12 mg 15 mg 30 mg	IT IT IT	2 2 2	8,22 8,22 8,22

**Figure 1.**
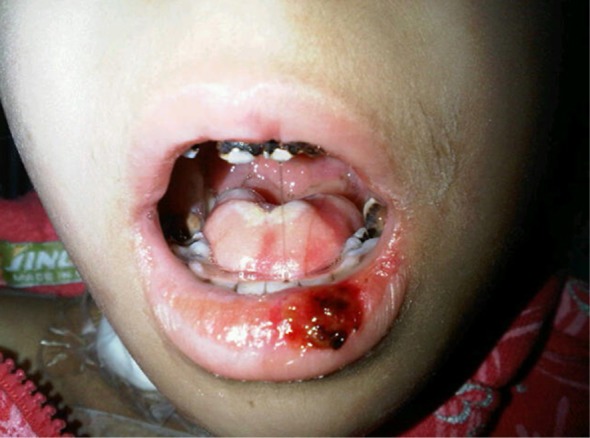
Patient’s oral mucosa showing erythema and necrosis at time of presentation with oral mucosal pain and fever.

On physical exam, the patient was sick-looking, lethargic and moderately pale. Her vital signs were: temperature = 38.7 ^o^C, HR = 120/min, RR = 24/min and BP = 90/60 mm Hg. Her weight was 12.2 kg (<3 ^rd^ % for her age) and her height was 110 cm (75% for her age). Her conjunctivae were mildly injected and her sclerae were non icteric. The oral cavity showed diffuse dental caries, severe mucosal erythema with whitish membranes and multiple necrotic and hemorrhagic lesions in the tongue, buccal mucosa and lower lip ( Figure 1). She was unable to drink fluids or eat solid food (WHO mucositis stage IV) ^1^. Her heart sounds were regular; a GII/VI systolic hemic murmur was heard all over the precordium. Lungs were clear and resonant. Abdomen was soft, non-tender with no hepatosplenomegaly or masses or ascites. There was no pathologic lymphadenopathy. Femoral pulses were symmetrical bilaterally and there was no peripheral edema. Neurologic exam, fundoscopy and skeletal exams were all normal. Laboratory tests upon admission showed severe neutropenia with moderate anemia and thrombocytopenia, increased prothrombin time (PT), partial thromboplastin time (PTT) and low fibrinogen ( Table 2).

**Table 2.** Laboratory results at presentation with fever and severe oral pain.

**Lab test**	**Lab results**	**Normal values**
White blood cell count	0.8 x 10 ^9^/L	4-11 x 10 ^9^/L
Hemoglobin	7.8 g/dl	11.5–12.5 g/dL
Platelets	28 x 10 ^9^/L	150–400 x 10 ^9^/L
Prothrombin time	55.5 sec	9.8–12.7 sec
INR	1.43	0.85–1.2
Partial thromboplastin time	54.2 sec	30–40 sec
Fibrinogen	<0.5 g/L	1.8–3.5 g/L
D-DIMER	158 µg/L	63.8–246.4 µg/L
Glucose	80,87 mg/dL	60–100 mg/dL
Sodium	129 mmol/L	135–145 mmol/L
Potassium	3.58 mmol/L	3.5–5.1 mmol/L
Calcium	7.92 mg/dL	8.5–10.5 mg/dL
Phosphorus	1.45 mg/dL	4–7 mg/dL
Magnesium	1.81 m/dL	1.71–2.29 mg/dL
SGOT	39.6 IU/L	0–50 IU/L
SGPT	37.2 IU/L	0–50 IU/L
GGT	30.2 IU/L	7–64 IU/L
Albumin/total protein	26.85 g/L/43.3 g/L	38–54g/L/60–80 g/L

INR = International normalized ratio

SGOT = serum glutamic oxaloacetic transaminase

SGPT = serum glutamic-pyruvic transaminase

GGT = *gamma-glutamyl transpeptidase*

The patient was started on intravenous cefepime (150 mg/Kg/d), vancomycin (60 mg/Kg/d), fluconazole (12 mg/Kg/d on day 1 followed by 6 mgs/Kg/d afterwards), topical mycostatin, 0.12% chlorhexidine mouthwash, adequate hydration and IV analgesia (perfelgan 15 mg/Kg given q6 hours). She also received vitamin K, platelet transfusion and fresh frozen plasma. On hospitalization day 3, the patient was still febrile with progressive mucositis and no oral intake. Micafungin 2.5 mg/Kg/day was started and fluconazole was discontinued. Total parenteral nutrition (TPN) was also initiated and packed red blood cell and platelet transfusions were continued as needed. On day 4, admission cultures of the lesions of the tongue and buccal mucosa grew *Candida albicans* and coagulase-negative staphylococci while blood culture was negative.

**Figure 2.**
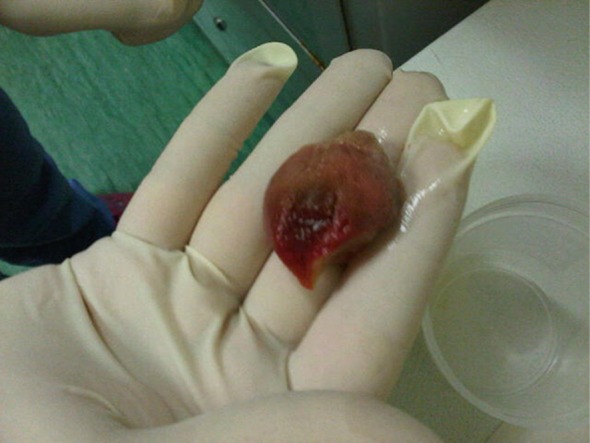
Shedding off the ventral part of the tongue.

The patient was steadily but slowly improving when on hospitalization day 6, she had an unexpected spontaneous shedding of the ventral part of her tongue ( Figure 2). Pathological analysis of the detached tongue confirmed necrosis and bacterial and fungal cultures were negative. Her speech and feeding were further impeded by this trauma. The patient was continued on IV imipenem (100 mg/Kg/d), vancomycin (60 mg/Kg/d), amikacin (20 mg/Kg/d) and fluconazole (12 mg/Kg/day day 1 followed by 6 mgs/Kg/day afterwards) antimicrobials and TPN. A psychology consultation was offered to the parents and the patient. On hospitalization day 13, her mucositis resolved and her oral food and fluid intake gradually recovered. Chemotherapy was initiated on hospitalization day 17 with no complications and the patient was discharged on day 22 in good condition. The family was instructed to adhere to the recommended daily oral care regimen consisting of topical mycostatin (100,000 unit/g applied BID), 0.12% chlorhexidine mouthwash, gentle tooth brushing and adequate oral hydration.

**Figure 3.**
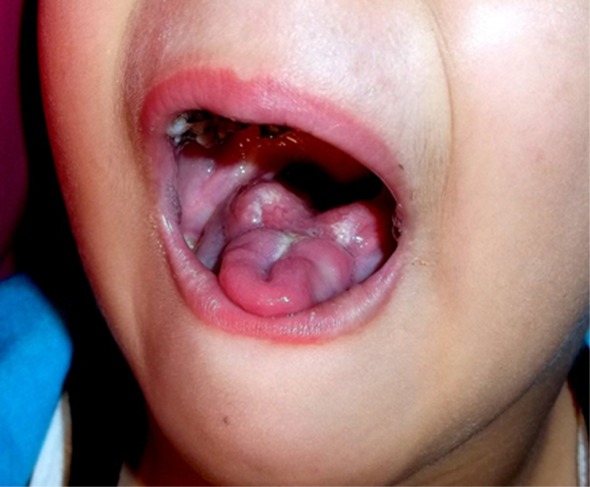
Tongue recovery 8 months after management.

Eight months after the incident, the patient recovered her ability to undergo oral maneuvers and her articulation steadily and progressively improved. Her tongue underwent compensatory hypertrophy ( Figure 3). She continues, though, to have slightly unintelligible speech at times. Currently, the patient is in complete remission and receiving her maintenance chemotherapy.

## Discussion

Leukemia and its treatment can adversely affect oral health. Leukemic cells are capable of infiltrating the gingiva and the deeper periodontal tissues resulting in local ulceration and infection. Mucosal cells are susceptible to chemotherapy due to their high mitotic rate ^2^. Oral mucositis is the most frequent and debilitating complication of chemotherapy in children with cancer and can be associated with serious morbidities and increased risk of infection ^2– 4^.

**Figure 4.**
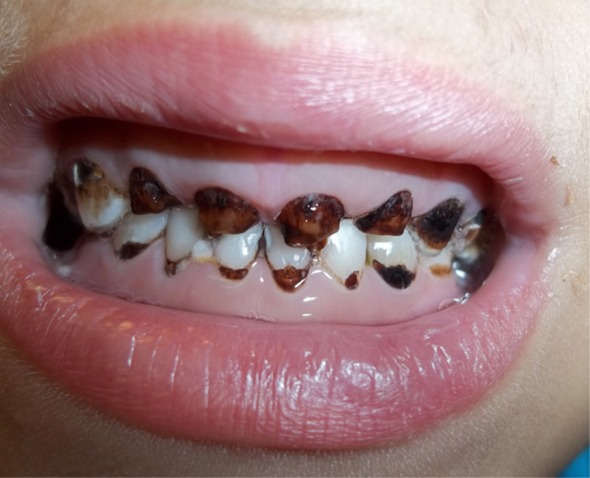
Dental caries at time of acute lymphoblastic leukemia diagnosis.

The decreased salivary flow rate, salivary pH and total salivary antioxidant levels in leukemic children compared to controls can lead to further deterioration in their oral health status, gingival status and increased dental caries ^3, 4^. Our patient suffered from poor dental hygiene and severe dental caries when diagnosed with pre-B ALL ( Figure 4). Oxidative stress per se may result in the onset of inflammatory oral pathologies. Saliva constitutes the first line of defense against free radical-mediated oxidative stress ^4^. The biological manifestations of mucositis include a surge in lipopolysaccharides or endotoxins which activate macrophages and other mononuclear cells leading to production of nitric oxide, tumor necrosis factor (TNF), interleukin-6 (IL-6) and other cytokines ^5, 6^.

To our knowledge, this is the first report of very severe chemotherapy-induced mucositis leading to shedding of a part of the tongue. We hypothesize other factors in this patient may have led to this fulminant and unique clinical presentation. The poor oral hygiene and the advanced dental caries the patient had at the time of ALL diagnosis could have contributed to the flare up of chemotherapy induced mucositis and to the shedding of part of the tongue. This hypothesis conforms to the previously reported association of exacerbated oral mucositis with trauma from the teeth ^4^. The degree of severity leading to tongue shedding in this patient was, however, remarkable. Notably, the tongue recovery and hypertrophic sublingual tissue seen in this patient 8 months after the incident are not unexpected and are a result of intrinsic tissue compensatory mechanisms which aided the patient in using her tongue for eating and speaking.

Children receiving chemotherapy should have critical and regular assessment of their oral mucosa before, during and after treatment. There are several oral assessment rating scales that can be used in cancer patients receiving chemotherapy such as the Oral Assessment Guide (OAG), Oral Mucositis Index (OMI) and Oral Mucositis Assessment Scale (OMAS) ^7^. While OAG addresses the oral functionality and tissue keratinization, the OMAS and OMI focus on the degree of tissue abrogation ^7^.

Careful oral management of children with cancer is critical and aims at preventing and treating mucositis and secondary infections as well as relieving pain and improving quality of life. An oral care protocol consisting of daily mouth rinsing with 0.12% chlorhexidine and tooth-brushing has been found to significantly decrease the incidence of ulcerative lesions, severity of oral mucositis and the related pain intensity compared to controls in pediatric cancer patients ^8^. In infants and very young children who are unable to rinse, care-givers need to use the chlorhexidine or its equivalent as an oral swab.

## Conclusion

This case represents a very severe chemotherapy-induced mucositis leading to a unique and previously unreported complication in a child with leukemia. It illustrates the fact that critical and regular assessment of the oral mucosa and proper dental care and oral hygiene are mandatory in all pediatric patients receiving chemotherapy. Physicians caring for children with cancer need to be aware of this rare complication and educate families about the benefits and modalities of optimal oral hygiene and the need to immediately report to their child’s health provider for mouth pain and or lesions. Additionally, vigilant treatment of mucositis needs to be instituted without delay in this high risk patient population. Such a preventive and therapeutic approach may prevent life threatening oral and systemic complications, promote mucosal healing and potentiate the hypertrophic compensatory action of linguinal cells thereby ensuring rapid and complete recovery.

## Consent

Written informed consent for publication of clinical details and clinical images was obtained from the patient’s legal guardian.
