# Supplementary material for: A rare aggravation of severe mucositis post chemotherapy in a child with acute lymphoblastic leukemia
Source: F1000Res. 2013 Sep 24;2:196. [Version 1] doi: 10.12688/f1000research.2-196.v1 (PMC3790598; doi:10.12688/f1000research.2-196.v1)
Supplement: Peer review history

# Peer review: Referee response for version 1

**Published:** 2013-09-25

**Authors:** Jan Stary

**Affiliations:** 1 Department of Pediatric Hematology and Oncology, University Hospital Motol, Prague, Czech Republic

The authors present the unusually severe complication of mucositis developed during the induction treatment of acute lymphoblastic leukemia. They stress the need for education of family and patients about the importance of oral hygiene during the intensive chemotherapy. The paper is well written and documented.

I have read this submission. I believe that I have an appropriate level of expertise to confirm that it is of an acceptable scientific standard.

# Peer review: Referee response for version 1

**Published:** 2013-09-24

**Authors:** Bassem Razzouk

**Affiliations:** 1 Children's Center for Cancer and Blood Diseases, Peyton Manning Children's Hospital, Indianapolis, Indiana, USA

This is a very well written case report about severe oral mucositis in a patient with ALL receiving induction therapy. The existing severe dental caries present at the diagnosis of ALL most likely contributed to the severity of the condition, which was managed appropriately and aggressively.

I have read this submission. I believe that I have an appropriate level of expertise to confirm that it is of an acceptable scientific standard.
